# Use of geographic information systems to assess the error associated with the use of place of residence in injury research

**DOI:** 10.1186/s40621-015-0059-y

**Published:** 2015-11-02

**Authors:** Ofer Amram, Nadine Schuurman, Natalie L. Yanchar, Ian Pike, Michael Friger, Donald Griesdale

**Affiliations:** 1 Department of Geography, Simon Fraser University, 8888 University Drive, Burnaby, BC Canada; 2 Department of Surgery, Dalhousie University, Halifax, NS Canada; 3 Department of Pediatrics, Faculty of Medicine, University of British Columbia, Vancouver, Canada; 4 BC Injury Research and Prevention Unit, Child and Family Research Institute, BC Children’s Hospital, Vancouver, Canada; 5 Faculty of Health Sciences, Ben-Gurion University, Beer Sheva, Israel; 6 Department of Anesthesiology, Pharmacology & Therapeutics, University of British Columbia, Vancouver, Canada

**Keywords:** Injury hotspot, Access to trauma systems, Locational error, Geographic information systems

## Abstract

**Background:**

In any spatial research, the use of accurate location data is critical to the reliability of the results. Unfortunately, however, many of the administrative data sets used in injury research do not include the location at which the injury takes place. The aim of this paper is to examine the error associated with using place of residence as opposed to place of injury when identifying injury hotspots and hospital access.

**Methods:**

Traumatic Brian Injury (TBI) data from the BC Trauma Registry (BCTR) was used to identify all TBI patients admitted to BC hospitals between January 2000 and March 2013. In order to estimate how locational error impacts the identification of injury hotspots, the data was aggregated to the level of dissemination area (DA) and census tract (CT) and a linear regression was performed using place of residence as a predictor for place of injury.

In order to assess the impact of locational error in studies examining hospital access, an analysis of the driving time between place of injury and place of residence and the difference in driving time between place of residence and the treatment hospital, and place of injury and the same hospital was conducted.

**Results:**

The driving time analysis indicated that 73.3 % of the injuries occurred within 5 min of place of residence, 11.2 % between five and ten minutes and 15.5 % over 20 min. Misclassification error occurs at both the DA and CT level. The residual map of the DA clearly shows more detailed misclassification.

As expected, the driving time between place of residence and place of injury and the difference between these same two locations and the treatment hospital share a positive relationship. In fact, the larger the distance was between the two locations, the larger the error was when estimating access to hospital.

**Conclusions:**

Our results highlight the need for more systematic recording of place of injury as this will allow researchers to more accurately pinpoint where injuries occur. It will also allow researchers to identify the causes of these injuries and to determine how these injuries might be prevented.

## Background

In the past several years, a number of epidemiological injury studies have used place of residence as a proxy for place of injury (Lawson et al. 2013a; Yiannakoulias et al. 2002; Cubbin et al. 2000; Lawson et al. 2015; Johnson and Lu 2011; Gruenewald et al. 2006). Using residence instead of place has been assumed to be accurate and potential locational error-and its effects on certainty-has yet to be investigated in detail. However, locational error impacts the accuracy of findings which can, in turn, affect injury prevention strategies. In any spatial epidemiological research, the use of accurate location data is critical to the reliability of the results. In injury research this is particularly important because the analysis and policy implications are often dependent upon knowing exactly where an injury occurred.

Unfortunately, however, many of the administrative data sets used in injury research do not include the location at which the injury takes place. Instead such data sets are typically restricted to locational data based on place of residence (normally at the postal code or zip code level) (Information CIfH. National/Ontario Trauma Registry Minimum Data Set CIHI 2014). As a result, researchers have no choice but to use place of residence in their analysis although this clearly introduces error within the data. Furthermore, in many cases, place of residence at the census level is also used to establish the socio economic status of the person injured and to draw links between socio economic status and injury mechanism (e.g., older houses, less well maintained playground equipment, etc.), however as injuries may take place in areas that do not reflect the same socio economic status as place of residence, this can also lead to error (it is important to note that SES also varies considerably within geographic areas, and even knowing place of residence at the census level is not guarantee that SES is the same as other individuals in the DA or CT). While there are many valid reasons to include place of residence in injury research, it is important that researchers be aware of the error that may result in using it as a proxy for place of injury.

The use of Geographic Information Systems (GIS) and spatial data in injury research has become more common in recent years (Bell and Schuurman 2010; Edelman 2007; Geurts et al. 2005; Lawson et al. 2013b). Over the past two decades, advances have also been made in data acquisition and spatial analysis techniques, allowing researchers to conduct more advanced spatial analysis (Graham et al. 2004). As a result, GIS is now more commonly used within injury and trauma systems research to determine the relationship between trauma centre access and injury outcome or to identify injury hotspots (geographic area of significant number of injuries) and their causes (Hameed et al. 2010; Nirula et al. 2010; Branas et al. 2005; Sampalis et al. 1997; Härtl et al. 2006). GIS also plays an important role in mapping the role of external environmental factors (e.g., playgrounds with faulty equipment) in order to establish prevention methods or remove hazards that may cause injuries (Catherine et al. 2004; Cubbin et al. 2000; Faelker et al. 2000; Williams et al. 1997).

Using GIS and spatial analysis methods, the aim of this paper is to examine the error associated with using place of residence as opposed to place of injury in injury research. Specifically, the paper will examine how this error affects specific age groups (i.e., children and school-age youth, working adults and adults over 65) and classification of injury mechanisms. In so doing, the paper is also meant to assist those researchers who must use place of residence to understand the impact that this error may have upon their results, and to suggest the means of minimizing its effect.

## Methods

Data Preparation

Traumatic Brian Injury (TBI) data from the BC Trauma Registry (BCTR) was used to identify all TBI patients admitted to BC hospitals between January 2000 and March 2013. Geographic information within the TBI dataset includes the hospital where the patient was treated, the patient’s place of residence and, when available, the place where the injury occurred, all at six-digit postal codes. All postal code variables were geocoded using the Statistics Canada Postal Code Conversion File (PCCF). Other variables collected included patient age and injury mechanism.

To assess the locational error between place of residence and place of injury, a calculation of driving time was made between these two locations. This calculation was done for five different age groups (0–4, 5–13, 14–18, 19–64 and 65 and older) as travel behaviours tend to vary throughout these age groups. For example, while small children spend much of their day at home, older children will spend a good part of their day at school (also typically close to home). Working adults, on the other hand, tend to travel somewhat further from home during the day while retired persons (65 or older) also tend to be at or closer to home during the day (Santos et al. 2011). The driving time calculation was made using the ArcGIS Network Analyst. This tool allows for relatively accurate estimations of travel time in that it provides turn-by-turn calculations while taking into account road speed limits. A more detailed explanation of this methodology can be found in previous publications (Cinnamon et al. 2008; Schuurman et al. 2010). The DMTI road data set was used to supply the road network data as it is suitable for use with the Network Analyst extension.

**Fig. 1:**
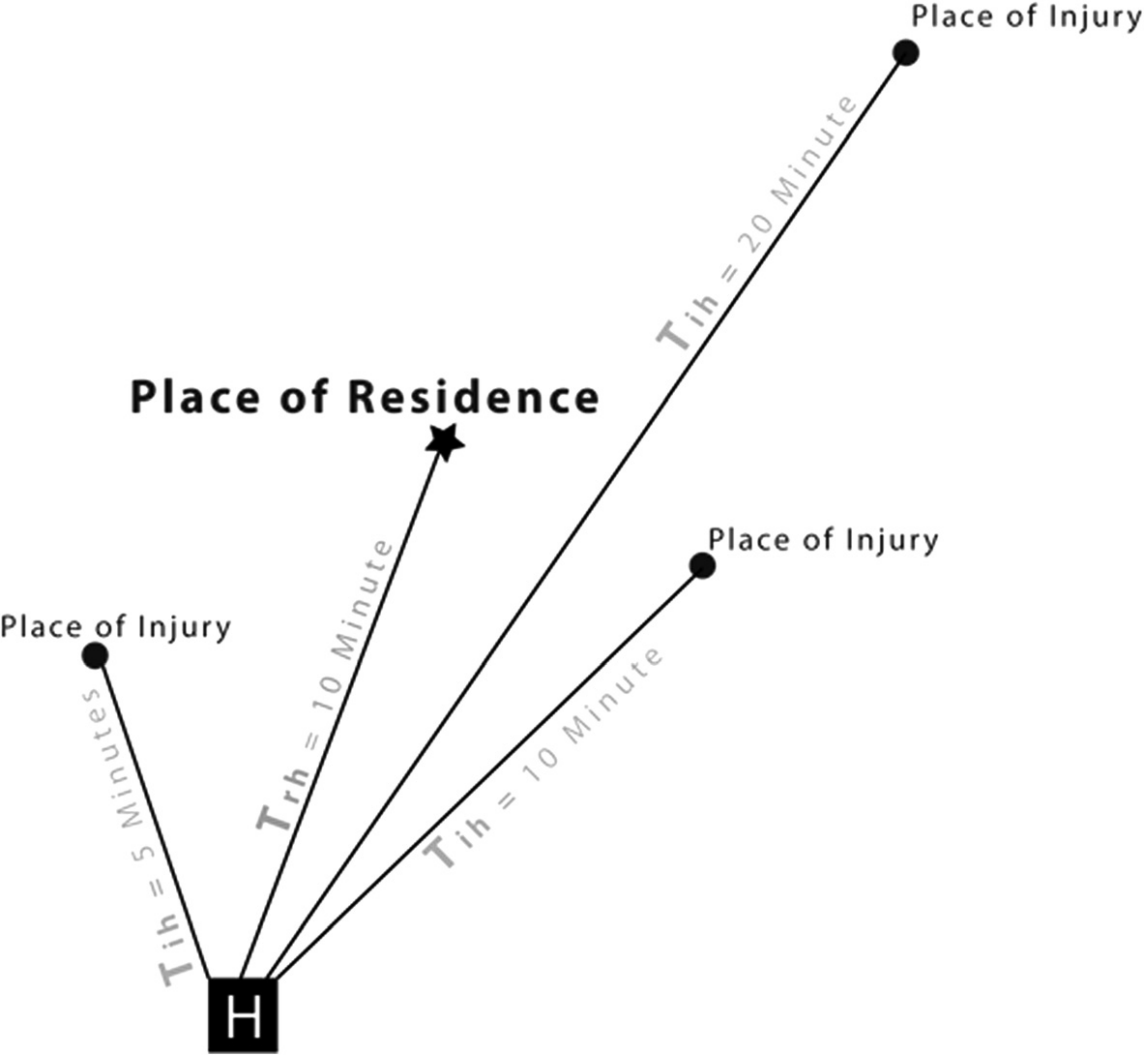
Illustrating locational error in driving time to the hospital. Shows how using place of residence as a proxy for place of injury can cause various errors in calculating driving time to hospital

While the assessment of the locational error affecting injury hotspots is relatively straightforward, the identification of locational error as it relates to hospital access is complex. This is because it requires examination of three separate driving time calculations: the first calculation describes driving time between place of residence and the hospital (T_rh_); the second between place of injury and the hospital(T_ih_); and the third calculation identifies the locational error (driving time) between place of injury and place of residence. In this situation, the third calculation (T_ir_) is not necessarily equivalent to the difference between the first and second calculations, because the place of injury may be much closer to the hospital than the place of residence or vice versa. Similarly, the place of injury could be in an entirely different location but have approximately the same driving time to the hospital (Fig. 1). In order to assess the locational error within the data, a calculation was made of the difference in driving times between both place of residence and the hospital where the patient was treated and place of injury and the same hospital (T_rh-_T_ih_). For this analysis, only patients transported directly to hospital by automobile were selected. All hospitals within BC that accepted these patients had their data included in the analysis.

### Estimating error for hotspot identification

In order to estimate how locational error impacts the identification of injury hotspots, the data was aggregated to the level of dissemination area (DA) and census tract (CT) and a linear regression was performed using place of residence as a predictor for place of injury. A residual map (mapping of the model residuals) was then created to highlight areas of under and over prediction at both the DA and CT level. As the DA is the smallest census unit at which census variables can be joined, they are often used to calculate disease rates. CT’s, on the other hand are much larger in size and are often used as a proxy for neighborhood in epidemiological studies (Kohen et al. 2002; Coulton et al. 2001). Only areas within greater Vancouver were used when examining errors in the identification of hotspots. This is because most of the areas outside greater Vancouver are primarily rural in nature and are comprised of large geographical administrative polygons (both CT’s and DA’s).

### Estimating error in access to trauma center studies

In order to assess the impact of locational error in studies examining hospital access, driving times were calculated between both place of residence and the hospital (T_rh_), and place of injury and the same hospital (T_ih_). An analysis of the driving time between place of injury and place of residence (T_ir_) and the difference in driving time between place of residence and the hospital and place of injury and the hospital was then conducted (T_rh_-T_ih_).

## Results

### Injury hotspots

**Fig. 2:**
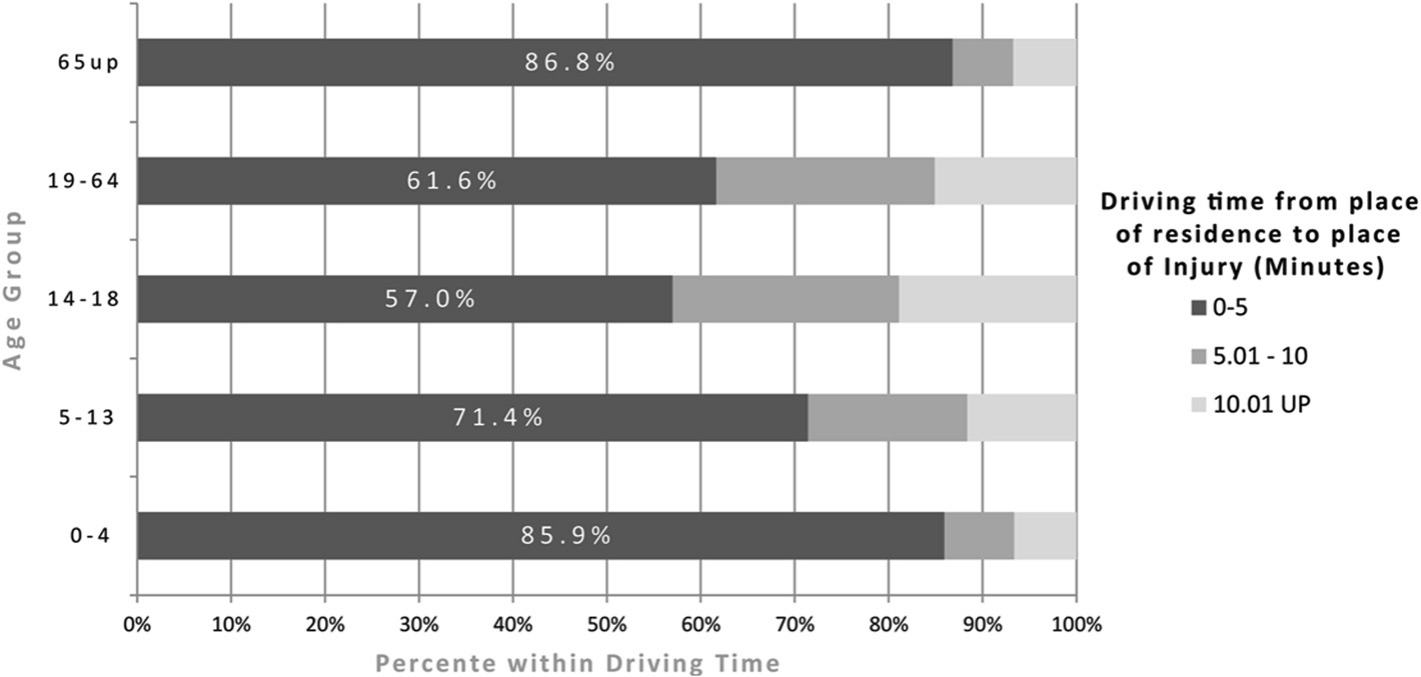
Driving time from place of residence to place of injury by age group. The graph indicates that 85 % of the injuries for those 65 and older occur within 5 min driving time of their place of residence

**Fig. 3:**
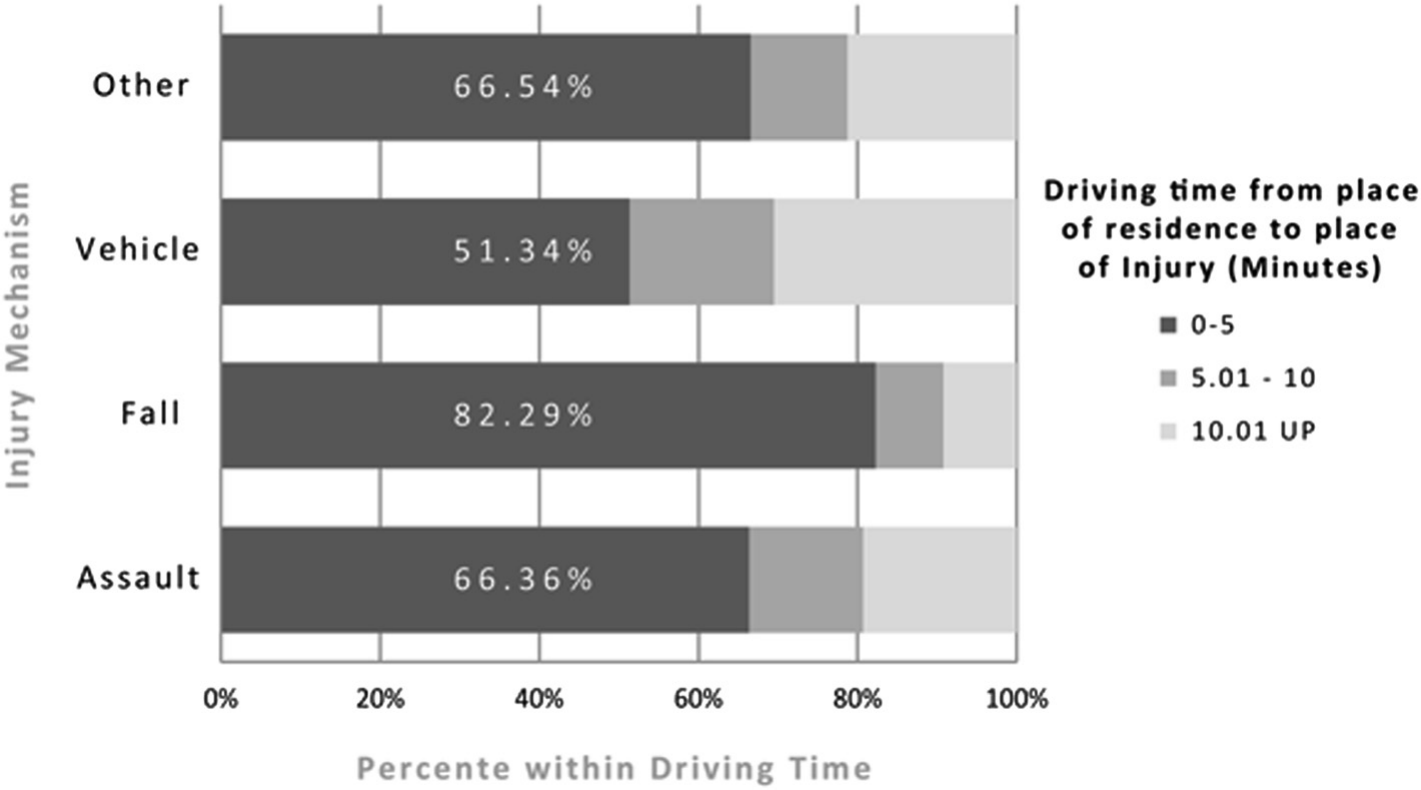
Driving time from place of residence to place of injury by injury mechanism. The figure indicates that injuries resulting from a fall tend to be closer to home while injuries resulting from a MVC tend to occur further away

The injury dataset in this analysis included 14,005 cases of TBI injury for which 7368 had complete postal code data on place of residence and place of injury (52 %) and therefore met the inclusion criteria for the study. Of the cases included, 73.3 % had injuries occurring within 5 min driving time of place of residence (T_ir_), 11.2 % between five and ten minutes and 15.5 % over 20 min. When the data was stratified by age group, the oldest (64 and up) and youngest (0–4) populations had the highest percentage (86.8 and 85.9 % respectively) of injuries that occurred within five minutes driving time of place of residence (T_ir_), while those 14–18 years of age had the lowest percentage of injuries within this same driving time range (Fig. 2). The data also showed trends in terms of injury mechanism. For example, 61.6 % of motor vehicle injuries occurred within 5 min of place of residence in contrast to 82.3 % of falls (Fig. 3).

**Fig. 4:**
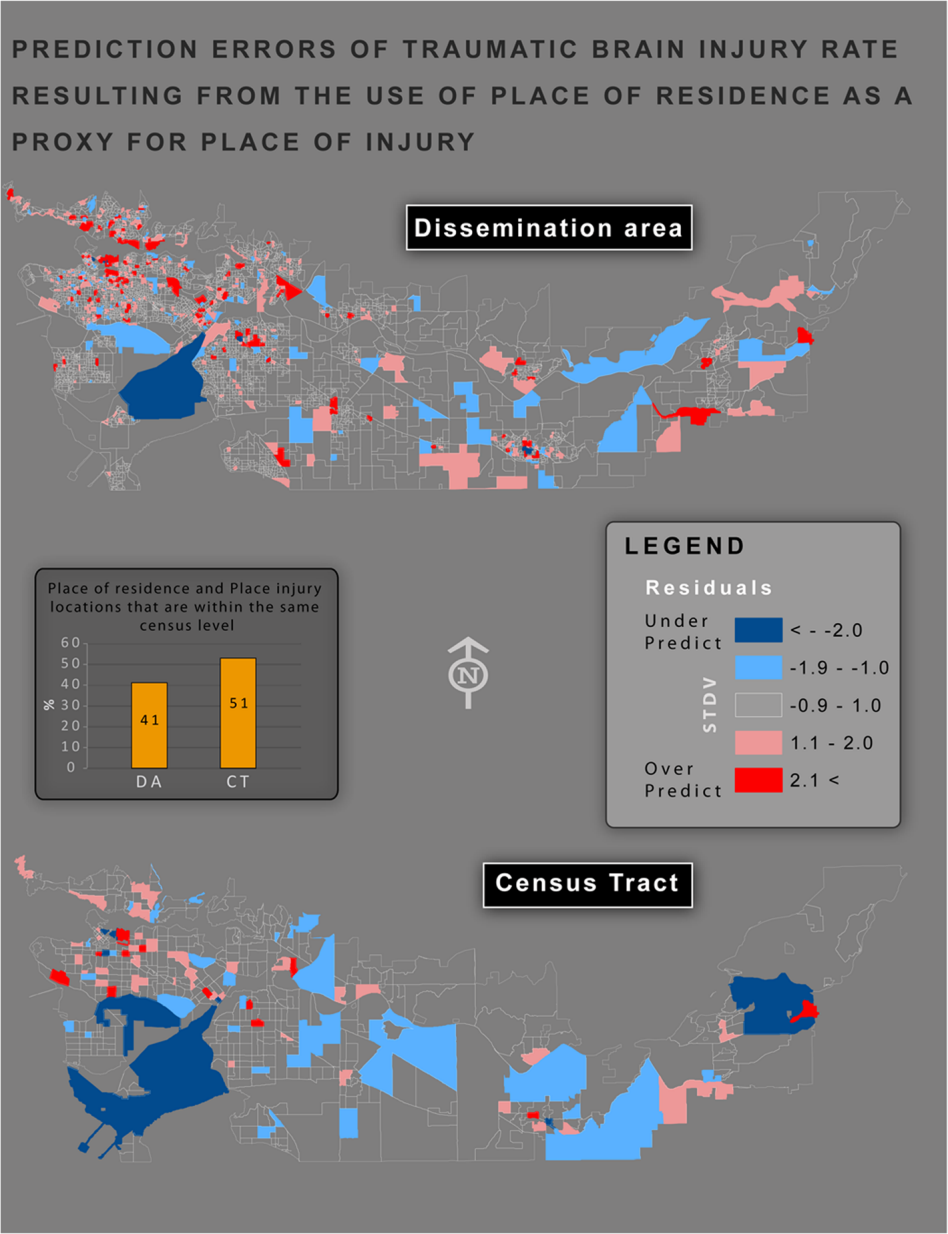
Shows the locational error at both the DA and CT level. The DA map clearly shows more detailed misclassification. This is because the DA’s smaller size makes it less common for both place of injury and place of residence to fall within the same area

Mapping injuries based on place of residence also results in error in the identification of injury hotspots. Figure 4 demonstrates the error over space at both the DA and CT level of TBIs within Greater Vancouver (include 3845 injuries based on location of injury). The residual map of the DA clearly shows more detailed misclassification. This is because the DA’s smaller size makes it less common for both place of injury and place of residence to fall within the same area (Fig. 4).

### Access to hospital

This analysis includes data from 636 cases that were transported to hospital via motor vehicle. In this particular dataset, the mean, median and interquartile measures of hospital driving time were almost identical from both place of residence and place of injury (Table 1). As expected, the driving time between place of residence to place of injury (T_ir_) and the difference between these same two locations and the hospital (T_rh_-T_ih_) share a positive relationship (Table 2). The greater the distance between the two locations, the greater the error when estimating driving time to the hospital.

**Table 1:** Descriptive statistics of driving time to the hospital from both place of residence and place of injury

	Driving time to hospital (Minutes)				
	Q1	Q3	Interquartile range	Mean	Median
Place of residence	12.2	103.2	91.0	96.4	40.8
Place of injury	11.5	100.6	89.1	96.7	42.3

**Table 2:** Driving time between place of injury and place of residence

	T_rh_-T_ih_: Difference in driving time between place of residence and hospital and place of injury and hospital (minutes)	
T_ir_: Driving time between place of injury and place of residence (minutes)	Mean	Median
0–10	0.9	0.1
10.1–20	8.7	9.4
20up	70.8	42.0

Shows that as the driving time between place of injury and place of residence increases, the error in driving time to the hospital also increases. The sharp difference in error indicates that the use of place of residence as a proxy for place of injury is reliable only with case populations where the distance between place of residence and place of injury is minimal (eg elderly, falls)

## Discussion

This study examines how using place of residence as a proxy for place of injury may result in errors in the description of injury hotspots and the measurement of trauma center access. Using GIS, this study highlights not only the numeric value of the error but also the spatial distribution of the error over space and provides valuable insight into how injury location may impact analysis and consequently the results of injury studies. Using highly accurate locational data for both place of injury and place of residence, this analysis examines the error in using place of residence as a proxy for place of injury. In addition, by visualizing how errors in cluster detection can propagate, this paper shows how the use of place or residence as a proxy for place of injury can easily lead to errors in cluster identification. This is the first study to use highly accurate data and to take this approach to draw attention to this subject. In addition, this paper also examines how locational error may impact access to trauma centres. In an era where the ubiquitous availability of GPS allows for the collection of extremely accurate location information, there is little reason to forgo the collection of specific injury location data, particularly given the importance location has on injury occurrence and outcome in terms of both space (distance to trauma care) and place (location of faulty playgrounds and unsafe neighborhoods).

The results of this study correspond with several other studies that indicate that injuries tend to take place in areas close to the patient’s place of residence. However, our hotspot analysis indicates that, at the city or regional scale, this relationship is not so clear. For example, only 41 % of the injury locations in this study fell within the same DA as did the patient’s place of residence. This figure jumps to over 50 % for CT’s indicating that the use of DA or CT level scale for hotspot detection may be problematic. One of the most comprehensive studies of this type was conducted by Myers et al. across the United States (Myers et al. 2011). Using over a million cases of death from injury, Myers concluded that the majority of injuries tend to occur within close proximity to place of residence. However, as noted in the study’s limitations section, the use of a county-level scale to determine whether an injury occurred close to home (i.e., if both place of residence and place of injury occurred in the same county it was considered in close proximity), is less precise than measures using address or zip code (Myers et al. 2011). Although our study uses a smaller dataset (with 7000 injuries the dataset is still large enough to provide detailed insight), it includes highly accurate postal code level location information and therefore provides more detail on the proximity of the two locations. Another study that examined violent injury also found that injuries taking place during daytime hours had similar hotspots for both place of residence and place of injury, indicating that these violent injuries were occurring closer to home. At night however, the hotspots tended to shift to areas farther from home (Cusimano et al. 2010). This study however, analyzed the data at the CT rather than the DA level. A different study, using a much smaller sample (321 participants), and done in a rural area of Wales, found that 80 % of the participants’ injuries occurred within 10 miles of home (Palmer et al. 2005). In recent years, the increased use of highly accurate locational data has enhanced researchers’ ability both to identify injury hotspots and to understand the causes of injury at these locations. In addition, the use of more accurate location data has allowed for a better understanding of how access to trauma care impacts injury outcomes and how trauma systems can be optimized to provide better care. However, the use of place of residence rather than place of injury when conducting such research can hinder the ability to accurately assess the impact of injury. The results of this paper clearly show that using place of residence rather than place of injury increases the uncertainty in the identification of injury hotspots (showing hotspots where no hotspots actually existed and vice versa). This paper also indicated that place of residence can provide a good proxy for place of injury, but only when the place of injury and the place of residence are within 10 min driving time of one another. This may only apply to certain sub-populations (eg elderly and the children at younger age) or with certain mechanisms of injury (e.g., falls). For the dataset in this study, almost 80 % of TBIs occurred within 10 min of place of residence.

The results from both analyses indicate that reliability is affected by geographic scale (census tract vs dissemination area) when using place of residence as a proxy for place of injury. In the hotpots analysis, the use of more aggregated census boundaries, like census tract (CT), improved the reliability of the results. Using CT levels resulted in better classification of hotspots and higher numbers of injuries where both place of injury and place of residence were within the same level (51 % for CT compared to 41 % for DA). However, while the use of CT (or even more aggregated census levels) for hotspot identification may increase confidence in the results, it will also reduce the precision with which each specific place of injury can be identified. The choice of scale should also take into consideration the characteristics of the study area. Based on our results, the use of place of residence as a proxy for place of injury may be better suited for urban areas where major activities, like going to school and work, tend to be closer to home. On the other hand, studies that are global or national in level are better suited to larger scale measures like those used in Myer’s study (Myers et al. 2011).

Another area of consideration is the type of injury being studied. As mentioned, motor vehicle injuries tend to take place further from home than do other types of injuries. This is supported by a U.S transportation report that found that only 47 % of the motor vehicle injuries take place within 5 miles from home (Boyle et al. 2007). Our study also supports this, in that our comparison of injury mechanisms, found motor vehicle injuries took place furthest from home, with a median driving time of 5 min between place of residence and place of injury. Because there is such a large degree of error for motor vehicle injuries, it is more challenging to compensate for this through the aggregation of census boundaries.

### Study limitations

This study has limitations that may skew the results. First, the measurement of hospital driving time included within the study is based on the hospital at which the patient was treated. This measurement was then compared to the driving time distance between the patient’s place of residence and the same hospital. In reality, however, in cases where the patient was injured at or close to his/her home, the patient may have been transported to a different hospital, assuming it was closer or had different specializations. While the hospital closest to the patient’s place of residence could have been used in this study, this would have been based purely on assumption. Instead, the recorded hospital was used within the study. Second, the driving time calculations utilized do not take into account traffic information. As a result, the driving times are likely underestimated in urban areas where traffic is congested.

## Conclusions

To the best of our knowledge, this is the first study to provide an in-depth look at how using place of residence rather than place of injury may lead to error. Our results highlight the need for more systematic recording of place of injury as this will allow researchers to more accurately pinpoint where injuries occur, to identify the causes of these injuries and to determine how these injuries might be prevented.
